# Transport Granules Bound with Nuclear Cap Binding Protein and Exon Junction Complex Are Associated with Microtubules and Spatially Separated from eIF4E Granules and P Bodies in Human Neuronal Processes

**DOI:** 10.3389/fmolb.2017.00093

**Published:** 2017-12-22

**Authors:** Dan O. Wang, Kensuke Ninomiya, Chihiro Mori, Ayako Koyama, Martine Haan, Makoto Kitabatake, Masatoshi Hagiwara, Kazuhiro Chida, Shin-Ichiro Takahashi, Mutsuhito Ohno, Naoyuki Kataoka

**Affiliations:** ^1^Institute for Integrated Cell-Material Sciences (WPI-iCeMS), Kyoto University, Kyoto, Japan; ^2^K-CONNEX (Keihanshin Consortium for Fostering Next Generation of Global Leaders in Research), Kyoto, Japan; ^3^Institute for Virus research, Kyoto University, Kyoto, Japan; ^4^Laboratory of Anatomy and Developmental Biology, Kyoto University School of Medicine, Kyoto, Japan; ^5^Laboratory of Cell Regulation, Departments of Applied Animal Sciences and Applied Biological Chemistry Graduate School of Agriculture and Life Sciences, The University of Tokyo, Kyoto, Japan; ^6^Medical Innovation Center, Laboratory for Malignancy Control Research, Kyoto University Graduate School of Medicine, Kyoto, Japan; ^7^Medical Top Track Program, Medical Research Institute, Tokyo Dental and Medical University, Tokyo, Japan

**Keywords:** CBP80, Y14, exon junction complex, neuronal cells, RNA transport

## Abstract

RNA transport and regulated local translation play critically important roles in spatially restricting gene expression in neurons. Heterogeneous population of RNA granules serve as motile units to translocate, store, translate, and degrade mRNAs in the dendrites contain *cis*-elements and *trans*-acting factors such as RNA-binding proteins and microRNAs to convey stimulus-, transcript-specific local translation. Here we report a class of mRNA granules in human neuronal processes that are enriched in the nuclear cap-binding protein complex (CBC) and exon junction complex (EJC) core components, Y14 and eIF4AIII. These granules are physically associated with stabilized microtubules and are spatially segregated from eIF4E-enriched granules and P-bodies. The existence of mRNAs retaining both nuclear cap binding protein and EJC in the distal sites of neuronal processes suggests that some localized mRNAs have not yet undergone the “very first translation,” which contribute to the spatio-temporal regulation of gene expression.

## Introduction

Localization, local translation, and degradation of mRNAs play important roles in asymmetric cell division, differentiation, cell motility, synaptic plasticity, and oogenesis (St Johnston, 2005; Kiebler and Bassell, 2006; Besse and Ephrussi, 2008; Holt and Bullock, 2009; Martin and Ephrussi, 2009; Medioni et al., 2012), In neurons, mRNA localization and subsequent local protein synthesis contribute to neuronal functions such as axon guidance, synaptogenesis, synapse pruning, axonal regeneration, and synaptic plasticity (Sutton and Schuman, 2006; Wang et al., 2010; Perry and Fainzilber, 2014; Tom Dieck et al., 2014). Cytoplasmic localization of mRNAs is often conferred by both *cis*-elements in 3′-untranslated regions (3′-UTRs) and the *trans*-acting factors that bind to and mediate the function of these elements (Eliscovich et al., 2013; Mitchell and Parker, 2014). Through this interaction, mRNAs and their associative proteins form messenger ribonucleoprotein particles (mRNPs) that are actively transported along the cytoskeleton to intracellular destinations. Many transport granules have been found along microtubules to be localized and function in neuronal processes (St Johnston, 2005; Kiebler and Bassell, 2006). Interestingly, actins rather than microtubules are enriched at synapses and peripheral domain of growth cones where localized mRNAs are either translated or stored in a translationally silent state until signals such as synaptic activities and guidance cues release the translational repression (Waung et al., 2008; Wang et al., 2009; Buxbaum et al., 2014).

It has been shown that mRNPs are assembled in the nucleus and splicing affects later cytoplasmic fate of the spliced mRNA (Dreyfuss et al., 2002; Trcek et al., 2011; Muller-McNicoll and Neugebauer, 2013; Boehm and Gehring, 2016). One of the best-characterized examples is *oskar* mRNA which localizes to the posterior region of Drosophila oocytes (Besse and Ephrussi, 2008). In addition to the *cis*-elements in its 3′UTR and *trans*-acting factor Staufen, posterior localization of *oskar* mRNA requires deposition of exon junction complex (EJC) in its first exon through splicing (Hachet and Ephrussi, 2004). Consistent with this finding, core components of EJC (eIF4AIII, Barentsz, Tsunagi, and Mago nashi) are critical for *oskar* mRNA localization (Hachet and Ephrussi, 2001, 2004). The molecular link between splicing and mRNA localization/local translation has also been demonstrated in mammalian neurons. One of the EJC core factors, eIF4AIII, plays important roles in regulating dendritic mRNA transport and stability (Giorgi et al., 2007). It was demonstrated that eIF4AIII is associated with UPF1 in mRNP in dendrites of hippocampal neurons (Giorgi et al., 2007). EJC may regulate the stability of target mRNA through nonsense-mediated mRNA decay pathway (NMD). NMD pathway selectively degrades premature termination codon (PTC)-harboring mRNAs to prevent aberrant protein production, thus serves as an important quality control process of gene expression (Rehwinkel et al., 2006; Isken and Maquat, 2008; Hwang and Maquat, 2011; Popp and Maquat, 2013; Karousis et al., 2016). It has been assumed that this process occurs on newly synthesized mRNAs during a “very first” translation when the mRNA is associated with nuclear cap binding heterodimer complex CBP80-CBP20 (CBC) (Rehwinkel et al., 2006; Isken and Maquat, 2008; Hwang and Maquat, 2011; Popp and Maquat, 2013; Karousis et al., 2016). CBC and EJC associate and co-export with the target mRNA in the mRNP from nucleus to cytoplasm (Ohno et al., 1990; Izaurralde et al., 1994; Kataoka et al., 1994, 1995, 2000, 2001; Gorlich et al., 1996; Kim et al., 2001; Le Hir et al., 2001) until the very first translation removes EJCs within the coding region and CBC is subsequently replaced by eIF4E at the mRNA cap. A PTC situated >50–55 nt upstream from an EJC effectively recruits SMG-1-Upf1-eRF1-eRF3 complex (SURF) and subjects the mRNA to NMD (Kashima et al., 2006).

Neuronal mRNAs have been identified that are regulated by natural NMD pathways (Giorgi et al., 2007). One of these mRNAs is *Arc* mRNA, which is transported to synapses and translated upon neuronal activities (Steward et al., 1998; Giorgi et al., 2007). It was discovered that *Arc* mRNA is degraded via NMD. Loss of eIF4AIII or Upf1 resulted in accumulation of Arc and increased the amplitude of miniature excitatory post-synaptic currents (Giorgi et al., 2007). Selective targeting and accumulation of *Arc* mRNA at synapses is mediated by activity-dependent degradation of the mRNA throughout dendrites, which is blocked by inhibiting NMDAR activation or protein synthesis (Steward et al., 1998; Farris et al., 2014). In addition, Lsm1, an auxiliary factor for mRNA degradation, and human Stau2 proteins coincide with CBP80 in the dendrites of rodent neurons (di Penta et al., 2009; Fritzsche et al., 2013; Heraud-Farlow et al., 2013). These results suggest that the nuclear mRNP assembly undergoes translation-dependent remodeling in neuronal processes that regulate the localization and degradation of the target mRNA.

Here we have analyzed CBP80-bound mRNPs in differentiated SH-SY5Y cells and human iPS-derived dopaminergic neurons by raising specific monoclonal antibodies against CBP80. We report that CBP80 granules are enriched in the shaft regions of neurites of both SH-SY5Y and iPS-derived dopaminergic neurons. CBP80-granules do not contain eukaryotic translation Initiation Factor 4E (eIF4E) or P-body markers such as decapping enzyme Dcp1. Furthermore, EJC core component Y14/RBM8A frequently colocalizes with CBP80, but not with eIF4E. Our results strongly suggest that specific dendritic mRNAs are transported into distal sites without undergoing the very first translation and the corresponding mRNP remodeling. Exchange of CBC to eIF4E may take place locally when mRNPs are released from the microtubule network.

## Materials and methods

### Reagents

Retinoic acid (cat#53279-24), RPMI medium (cat#30264-14), DMEM medium (cat#08456-65) were obtained from Nacalai Tesque, Japan. Hoechst33342 was from Sigma-Aldrich. Recombinant human BDNF (cat#450-02), Cell-culture grade collagen (DME-02), penicillin-streptomycin (cat#15140-122) were purchased from Peprotech, Atelocell and Life Technology, respectively.

### Antibodies

Antibodies used for this study are as follows;

rabbit anti-eIF4E (1:200, sc-13963, Santa Cruz), rabbit anti-Tubulin (1:100 #2144 Cell Signaling), mouse anti-Tubulin (1:1000, T6793, Sigma), rabbit anti-Y14 (mono, 1:1000, and poly, 1:10, sc-134154, Santa Cruz), rabbit anti-Dcp1a (1:500, D5444, Sigma), rabbit anti-FMR1 (1:500, N-terminal (N), F3930, 1:500, C-terminal (C), F4055, Sigma), anti-Importin α (1:250 A484A, Bethyl Laboratories), anti-tyrosine hydroxylase (Millipore, cat#AB152), CF™488A dye for phalloidin staining (Biotium). Anti-eIF4AIII antibody was a kind gift from Dr. Akio Yamashita (Okada-Katsuhata et al., 2012) and used for immunostaining of cells at a dilution of 1:250.

### Preparation of CBP80 recombinant proteins and production of monoclonal antibodies against CBP80

The hCBP80 cDNA was amplified from human cDNA by PCR and cloned into pFastBac Htb digested with *Bam*HI and *Xho*I. The hCBP80 Bacmid clone was prepared using Bac-to-Bac Baculovirus Expression System (Life Technologies) and introduced to Sf9 cells by Cellfectin II transfection reagent (Life Technologies) according to the manufacturer's instruction. For protein production, Sf9 cells were infected at MOI = 5. After viral amplification for 3 days, the infected cells were harvested and used for the hCBP80 purification by Ni-NTA agarose (Qiagen). The final eluate was dialyzed against the storage buffer [10 mM Hepes (7.9), 200 mM NaCl, 20% Glycerol] and concentrated. Anti-hCBP80 mouse monoclonal antibodies were raised at Immuno-Biological Laboratories Co, Ltd (Fujioka, Japan).

### Cell culture and differentiation

SH-SY5Y cells were cultured in medium supplemented with antibiotics (100 U/mL streptomycin and 100 μg/mL penicillin; Sigma) and 10% fetal calf serum at 37°C in 5% CO_2_. For neuronal differentiation, the cells were cultured in RPMI1640 medium (Nacalai Tesque) on collagen-coated dishes (day 0) and treated with 10 μM retinoic acid from day 1 to 5. And then, the cells were washed three times with serum-free DMEM (Nacalai Tesque) and cultured in DMEM containing 50 ng/ml BDNF from day 5 to 9–11. If necessary, the cells were treated with nocodazole (10 ng/ml, for 30 min), cytochalasinD (25 ng/ml, for 30 min) or cycloheximide (100 ng/ml, for 2 h) before analyses.

### Immunoprecipitation of rNAs

SH-SY5Y cells were seeded into thirty plates of 10 cm collagen-coated dishes (BioCoat, CORNING). To obtain cytoplasmic fractions, cells were harvested after 4 days of BDNF treatment described above. Therefore, cells were washed twice with 10 ml of ice-cold PBS per plate, then 0.5 ml of Lysis buffer (10 mM Tris-Cl, pH 7.4, 100 mM NaCl, 2.5 mM MgCl_2_, 35 μg/ml digitonin) were added and the cells scraped from the plates with a Cell Lifter (CORNING) and harvested into a 50 ml tube (Thermo Scientific). After incubation for 5 min on ice, cells were disrupted by passage through 25G needles (TERUMO, Tokyo, Japan) four times. Centrifugation at 4,000 g briefly yielded a supernatant fraction that was further used for immunoprecipitation and designated the cytoplasmic fraction. In order to immobilize antibodies, 40 μl (bed volume) of the Protein A magnetic beads (Tamagawa Seiki, Nagano, Japan) were added per tube and washed twice with 0.5 ml of nuclease-free ice-cold PBS, followed by a wash with 0.5 ml of Lysis buffer. To each tube 0.5 ml of Lysis buffer and 20 μg of antibodies (Normal Mouse IgG (Santa-Cruz) for negative control or 38A1 for target CBP80 precipitation, respectively) were added. The tubes were incubated for 1 h at 4°C under rotation. After immobilization, the supernatant was removed and the beads transferred into 15 ml tubes. Eight milliliter of the cytoplasmic fraction were added to each tube and the mixture incubated at 4°C for 1 h under rotation. The supernatant was removed and the beads were washed 5 times with 0.5 ml of Lysis buffer. After wash, precipitated RNAs were eluted with TRIzol (Invitrogen) using the manufacturer's protocol and recovered by ethanol precipitation. After measuring the concentration, the RNAs were used for RT-PCR.

### RT-PCR analysis

For detection of UHG and GAPDH RNAs, total RNAs were recovered from cultured cells with TRIzol according to the manufacturer's instructions (Invitrogen). The RNAs were treated with RNase-free DNase (RQ1; Promega) according to the manufacturer's recommendation. First-strand cDNA was synthesized using reverse transcription (Prime Star, Takara Bio Inc., Japan) with random hexamers, and amplified by PCR. RT-PCR was performed with Ex Taq polymerase (Takara Bio Inc.). Cycle conditions were as follows: 94°C for 2 min; followed by 30 cycles (*UHG*) or 26 cycles (*GAPDH*) of 94°C denaturation for 10 s, 55°C (*UHG*) or 58°C (*GAPDH*) annealing for 30 s, and 72°C elongation for 30 s; with a final incubation at 72°C for 5 min in a PCR Thermal Cycler (BIOMETRA). PCR products were separated by electrophoresis and stained with ethidium bromide. Real-Time quantitative PCR reactions were performed with THUNDERBIRD SYBR qPCR Mix (TOYOBO, Japan) and analyzed by Step One Plus Real-Time PCR system (Applied Biosystems). The primers for RT-PCR and RT-qPCR are as follows;

UHG F, 5′-TCATTTTTCTACTGCTCGTGGATTTAC-3′,
UHG R, 5′-GTCTTGTCCCATTCCTTCACCA-3′,
GAPDH F, 5′-ATGAGAAGTATGACAACAGCCTCAAGAT-3′,
GAPDH R 5′-ATGAGTCCTTCCACGATACCAAAGTT-3′

As for immunoprecipitated RNAs, DNase treatment and first strand cDNA synthesis were carried out as described above. RT-PCR was also performed with Ex Taq polymerase (Takara Bio Inc.). Cycle conditions were as follows: 94°C for 2 min; followed by 30 cycles of 94°C denaturation for 10 s, 55°C annealing for 30 s, and 72°C elongation for 30 s; with a final incubation at 72°C for 2 min in a PCR Thermal Cycler (BIOMETRA). The PCR products were analyzed by agarose gel electrophoresis followed by ethidium bromide staining. The primers for RT-PCR are as follows; β-actin F, 5′-AGAGCTACGAGCTGCCTGAC-3′,

- β-actin R, 5′-AGCACTGTGTTGGCGTACAG-3′,
- MAP2ab F, 5′- GGATGAGTGGGGTTTAGTTGC-3′,
- MAP2ab R, 5′- AAAACTATCTTTGGCAGTAGCTG-3′,
- PSMB4 F, 5′-CCTCAGTCCTCGGCGTTAAG-3′,
- PSMB4 R, 5′-GCATGGTACTGTTGTTGACTCG-3′,
- GAPDH F, 5′-CACCCACTCCTCCACCTTTGA,
- GAPDH R, 5′-GTCCACCACCCTGTTGCTGTAG
- Ran F, 5′- AGCCCCAGGTCCAGTTCAAAC-3′,
- Ran R, 5′- ATGGCACACTGGGCTTGGATA-3′,
- Phgdh F, 5′- ATTGTCGGCCTCCTGAAAGA-3′,
- Phgdh R, 5′- TGAAGACAGCTCCATTGAGC-3′,
- TFIIB F, 5′- CCTACAGACTTCAAATTTGAC-3′,
- TFIIB R, 5′- TCCGTTTTGAAAAATGTTTTATTC-3′

For quantitation analysis, signals were measured by ImageJ software [U.S. National Institutes of Health, Bethesda (Schneider et al., 2012)].

### Sedimentation assay

Cultured cells were washed in ice-cold PBS(-) and scraped in sedimentation buffer [100 mM PIPES (pH 6.9), 1 mM MgCl_2_, 1 mM EDTA, 0.5% TritonX-100, 4 M glycerol] containing 5 μM taxol, 10 ng/ml nocodazole or 25 ng/ml cytochalasinD). The cells were homogenized by gently pipetting and centrifuged at 12,000 g for 5 min at 15°C. The supernatant was centrifuged again at 32,000 g for 30 min at 15°C. The supernatant (monomer or short polymer fraction of tubulin and actin) was diluted with the same volume of 2x SDS sample buffer. The pellet (polymer fraction) was rinsed in new sedimentation buffer and centrifuged at 32,000 g for 10 min at 15°C, and the pellet was dissolved in SDS sample buffer.

### iPS cell culture and differentiation

ReproNeuro DA kit containing frozen iPS cell vial (3 × 10^5^cells), coating medium, thawing medium, maturation medium, and additive A was purchased from ReproCell (cat# RCESD011) and dopaminergic neurons were derived following the manufacturer's instructions. Briefly, upon receiving the kit, cells were stored at −80°C until thawing. One day before thawing the cells, 40 wells of a 96-well plate were coated in 0.002% PLL/PBS solution at 4°C for at least 2 h. After rinsing twice with PLL/PBS, coating solution was added (50 μl well) and the plate was incubated in a 37°C incubator overnight. On the day of plating cells, first 100 μL additive A and 50 μL penicillin/streptomycin solution were added to 5 mL of maturation medium, and stored at 4°C. The cells were thawed by warming up the cell vial in a 37°C water bath and immediately transferred into 10 mL prewarmed thawing medium. Cells were spinned down at 350 g for 5 min and resuspended in 1.5 mL maturation medium. Cells were seeded at a density of 3.0 × 10^4^ cells per well. Cells were fed on days 3, 7, and 14 by replacing half the medium with fresh maturation medium. After 14 days, the cells were ready for assays. We monitored successful differentiation of the cells into dopaminergic neurons by immunostaining with an anti-tyrosine hydroxylase antibody. Thirty percentage of the differentiated neurons were dopaminergic neurons.

### Immunofluorescence staining and western blotting

Cells cultured on coverslips in 24-well format were fixed in 4% PFA/PBS for 15 min at room temperature and washed three times with DEPC/PBS. Fixed cells were permeabilized in 70% refrigerated EtOH on bench for 10 min and washed again by DEPC/PBS for three times. Permeabilized cells were incubated in 2% BSA/PBS solution for blocking and primary antibodies were applied and incubated in blocking solution for 1 h. After three washes with DEPC/PBS+0.1% Tween, secondary antibody was incubated in blocking solution in the dark for 1 h. Stained coverslips were washed three times in DEPC/PBS+0.1% Tween and mounted in ProLong mounting media with DAPI for imaging.

SDS-PAGE and western blot analysis were performed as previously described (Ninomiya et al., 2011). The signals were developed by chemiluminescence reaction using Chemi-Lumi One Super detection reagents (Nacalai Tesque, Japan) and detected with Image Quant LAS4000 (GE healthcare).

### *In situ* hybridization

*In situ* hybridization was performed basically as previously described (Ninomiya et al., 2011). To detect poly(A) mRNAs, biotinylated oligo dT(30) probe and FITC-conjugated streptavidin were used. Templates for MAP2ab or TFIIB probes were amplified from human brain cDNA by PCR using following primer sets;

MAP2ab (5′-GGATGAGTGGGGTTTAGTTGC-3′, 5′-AAAACTATCTTTGGCAGTAGCTG3′),
TFIIB (5′-CCTACAGACTTCAAATTTGAC-3′, 5′-TCCGTTTTGAAAAATGTTTTATTC-3′).

### Imaging and quantitative analysis

Fluorescence confocal images were acquired on LSM780 (Zeiss) with Plan-Apochromat 63x/1.40 Oil DIC M27 with an argon laser 488 nm: 0.9000% (ChS1: 491–604) and a red diode laser 633 nm: 1.5% (Ch2: 638–755), optical sectioning thickness: 30–100 μm, and the distance in neurites from the edge of cell bodies is between 20 to 100 μm. Immunoactive granules in neuronal processes were identified and counted by blind experimenters and colocalization was quantified as (# of granules containing concentrations of both A and B)/(# of granules containing concentrations of A and/or B).

## Results

### Human CBP80-specific monoclonal antibodies detect poly(A) RNA granules in the neuronal process of SH-SY5Y cells

To investigate local RNA regulation in human neurons, we used an *in vitro* differentiation model cell line of SH-SY5Y. Stepwise treatment with retinoic acid and BDNF caused the neuroblastoma cells to grow extended and branched neurites with apparent resemblance to neurons (Supplemental Figure 1A). In differentiated SH-SY5Y, mRNAs localized to neuronal processes (e.g., MAP2ab) and mRNAs restricted to soma (e.g., TFIIB) were detected by *in situ* hybridization. The number of puncta of MAP2ab mRNA detected in the processes of SH-SY5Y was almost six times higher than for TFIIB mRNA (Supplemental Figure 1B). Furthermore, cycloheximide treatment increased the expression level of natural NMD target genes such as U22 host gene (UHG) in differentiated SH-SY5Y cells, indicating that NMD is active in this cell model system (Figures S1C,D). Thus, we concluded that SH-SY5Y is a suitable human cellular model for investigating localized mRNA translation and natural NMD pathways.

**Figure 1:**
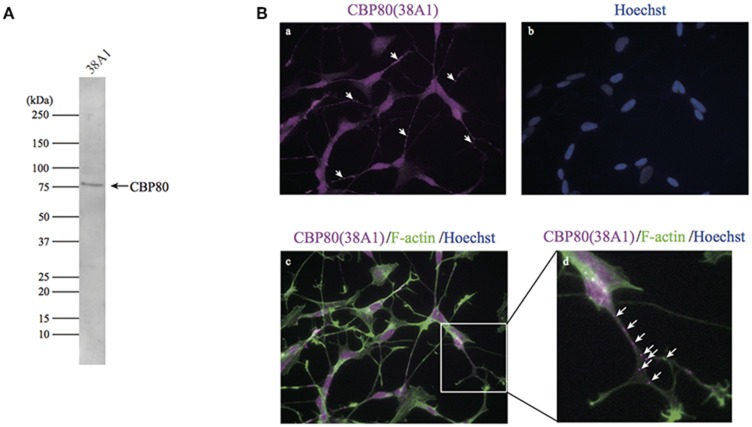
CBP80-positive puncta in differentiated SH-SY5Y cells. **(A)** Western blot analysis of SH-SY5Y lysate with monoclonal antibody 38A1 raised against human CBP80. **(B)** Microimages of SH-SY5Y cells stained with 38A1 **(a)** and Hoechst **(b)**. **(c)** Microimages of SH-SY5Y cells co-stained with 38A1 antibody (magenta) and Phalloidin-fluorescein (green). **(d)** Enlarged image of white box in **(c)**. Arrows indicate CBP80 granules in neuronal processes.

To determine cellular distribution of CBP80 (RNA cap binding complex component), we tried to prepare good monoclonal antibodies that work for western blotting, immunofluorescence and immunoprecipitation. We could produce a human CBP80-specific monoclonal antibody, 38A1, in mice using a full-length CBP80 protein. Western-blot analysis with this antibody detected a single band around 80 kDa in the total lysate of SH-SY5Y cells (Figure 1A). Fluorescence immunostaining with 38A1 revealed predominant fluorescence signals in the nucleus of SH-SY5Y cells, consistent with the role of CBP80 as a component of the early cap-binding complex of mRNA after RNA synthesis. In addition, fluorescent puncta were detected in the cytoplasm (Figures 1B,a). Furthermore, bright puncta were detected in the soma and neurites of differentiated SH-SY5Y cells, as shown through co-staining with 38A1 and actin (Figures 1B,d).

**Figure 2:**
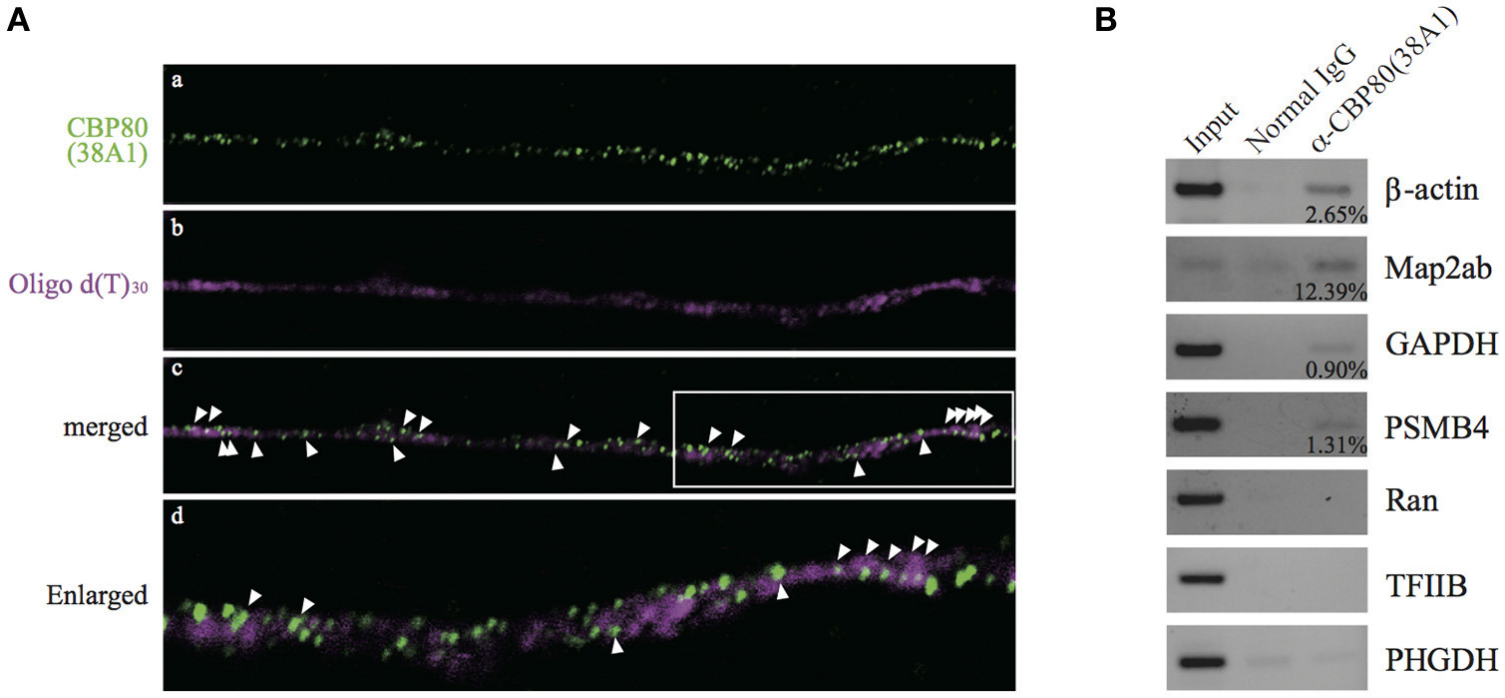
CBP80-positive puncta contain poly(A) RNA. **(A)** Microimages of differentiated SH-SY5Y cells stained with **(a)** 38A1 antibody against CBP80 (green) and **(b)** oligo d(T)_30_-fluorescein (magenta). The merged image is shown in **(c)**. Arrowheads point to fluorescent puncta that contain both CBP80 and poly(A). **(d)** Enlarged image of white box in **(c)**. **(B)** RT-PCR analyses of the mRNAs from the co-immunoprecipitation experiments. Input lanes contain 10% of total amount of RNA recovered from the cytoplasmic fractions used for RT-PCR. Normal mouse IgG was used as a control and anti-CBP80 antibody (38A1) was utilized for immunoprecipitation of RNP. Input lanes contain 10% of total input used for immunoprecipitation. The percentages of immunoprecipitated mRNAs by anti-CBP80 (38A1) are indicated in the figure.

In order to test whether the CBP80-positive granules along the neurites are mRNA RNPs or not, we performed *in situ* hybridization in the differentiated SH-SY5Y cells with oligo d(T)_30_ probe simultaneously with CBP80 antibody 38A1 immunostaining. Fluorescent signals from oligo d(T)_30_ were detected in some of CBP80 immunopositive puncta, suggesting that the CBC-bound granules contained poly(A) RNA (Figure 2A). The oligo d(T)_30_ signals were less punctate than those of anti-CBP80 antibody staining. It is likely this is caused by accessibility problem of oligo d(T) probe. To determine specific mRNA species associated with CBP80, we immunoprecipitated CBP80-RNA complexes from cytoplasmic fractions of differentiated SH-SY5Y cells. Reverse transcription and polymerase chain reaction (RT-PCR) were performed with gene-specific primers to amplify cDNA fragments of target genes. As shown in Figure 2B, anti-CBP80 antibody (38A1) precipitated β-actin, MAP2ab, PSMB4, and GAPDH mRNAs that are known to be localized in neurons (Giorgi et al., 2007; Taylor et al., 2009), although co-precipitation efficiencies varied among those mRNAs. In contrast, non-localizing mRNAs such as Ran, TFIIB, or PHGDH were not co-immunoprecipitated with CBP80. These results strongly suggest that CBP80-positive granules are transport granules containing localized mRNAs.

### CBP80-granules are associated with microtubules and spatially separated from eIF4E-granules

**Figure 3:**
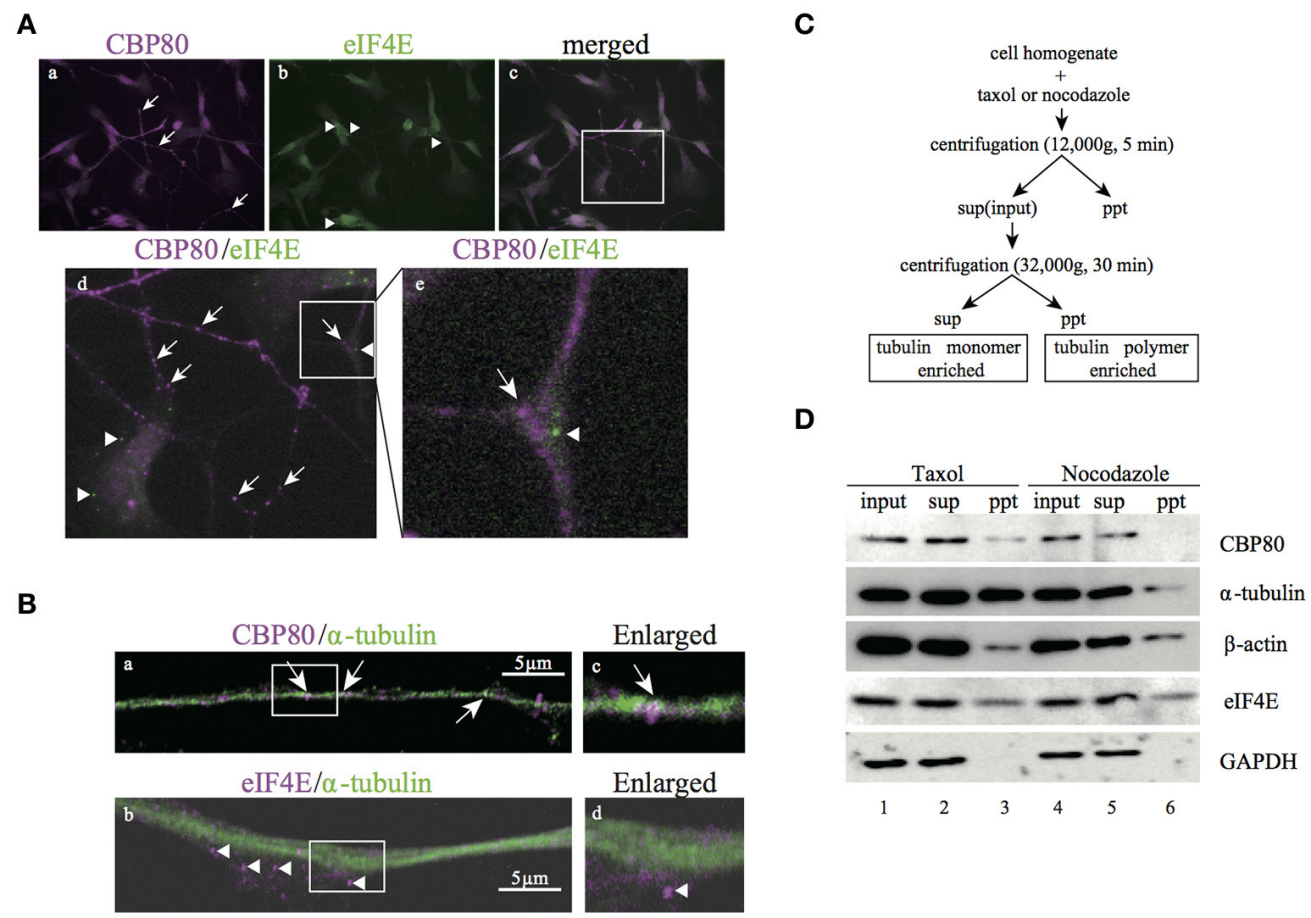
CBP80-RNPs colocalize with microtubules and are spatially segregated from eIF4E-RNPs. **(A)** Microimages of differentiated SH-SY5Y cells co-stained with antibodies against CBP80 (**a**, magenta), and eIF4E (**b**, green). The Merged image is shown in **(c)** and the part in the white box enlarged in **(d)** or **(e)**, respectively. Arrows point to CBP80-positive puncta and arrowheads point to eIF4E-positive puncta. (**d,e**) Enlarged images in the white box. Arrow and arrowhead point to CBP80-positive punctum and eIF4E-positive punctum, respectively. **(B)** Confocal images of SH-SY5Y neuronal processes double-stained with CBP80 (magenta) **(a)** or eIF4E (magenta) **(b)** and α-tubulin (green) antibodies. The enlarged images of **(a)** and **(b)** are shown in **(c)** and **(d)**, respectively. White arrows point to CBP80-positive puncta and arrowheads point to eIF4E-positive puncta. Scale bars, 5 μm. **(C)** The procedure of the biochemical fractionation assay for analysis of SH-SY5Y cells treated with taxol or nocodazole. sup: supernatant, ppt: precipitant. **(D)** Western blot analyses of CBP80, α-tubulin, β-actin, eIF4E, and GAPDH in different fractions.

The existence of mRNA granules enriched with CBP80 along the human neuronal processes raises the question whether these mRNA granules represent early steps in the mRNA remodeling process before the nuclear cap binding proteins are replaced by the cytoplasmic cap binding protein eIF4E. If so, these granules should be mostly different from those enriched with eIF4E proteins. In order to test this hypothesis, we double-stained differentiated SH-SY5Y cells with specific antibodies against CBP80 and eIF4E. As expected, eIF4E immunofluorescence was distributed to soma and neurites in puncta (Figures 3A,b). Colocalization analysis of eIF4E- and CBP80-positive puncta revealed that there was little overlap between the two RNP populations (Figure 3A,d,e, Supplemental Table 1), suggesting spatial segregation of mRNA granules containing mRNA transcripts capped by either CBP80 or eIF4E.

The spatial segregation of CBP80 from eIF4E-positive puncta suggests that the CBP80-positive puncta may represent an independent type of transport granules containing translationally repressed mRNAs. To test this, we first analyzed colocalization of CBP80 and tubulin and found that CBP80-positive puncta were distributed along tubulin-positive fibers (Figures 3B,a). In contrast, eIF4E-granules were localized to areas with less tubulin staining (Figures 3B,b). This localization pattern suggests that CBP80- but not eIF4E-granules are associated with microtubule bundles and transported along microtubules. To confirm this hypothesis, we took a biochemical approach to purify polymerized cellular cytoskeleton networks from differentiated SH-SY5Y cells based on sedimentation (Figure 3C). This centrifugation-based procedure allows polymerized heavy cytoskeleton networks such as the microtubule lattice to sediment in pellet together with their interacting factors. Treating the cell homogenate with Taxol specifically stabilizes microtubule polymerization, thus enriching the pellet of polymerized microtubules and factors attached. Conversely, nocodazole treatment destabilizes microtubules thus releases components such as α-tubulin monomers and associating proteins from pellets into the supernatant. Western blot analysis with antibodies raised against CBP80, α-tubulin, β-actin, and eIF4E showed that the signals of CBP80 and α-tubulin dramatically decreased in the pellet after nocodazole treatment, suggesting direct or indirect attachment of CBP80-granules to stabilized microtubules. In contrast, the concentration of β-actin and eIF4E in the pellet did not differ between taxol- and nocodazole-treated homogenates, indicating that these proteins do not interact with polymerized microtubules. In a control experiment, soluble protein GAPDH was only detected in the supernatant, demonstrating high purity of the insoluble cytoskeleton network in this assay (Figure 3D).

**Figure 4:**
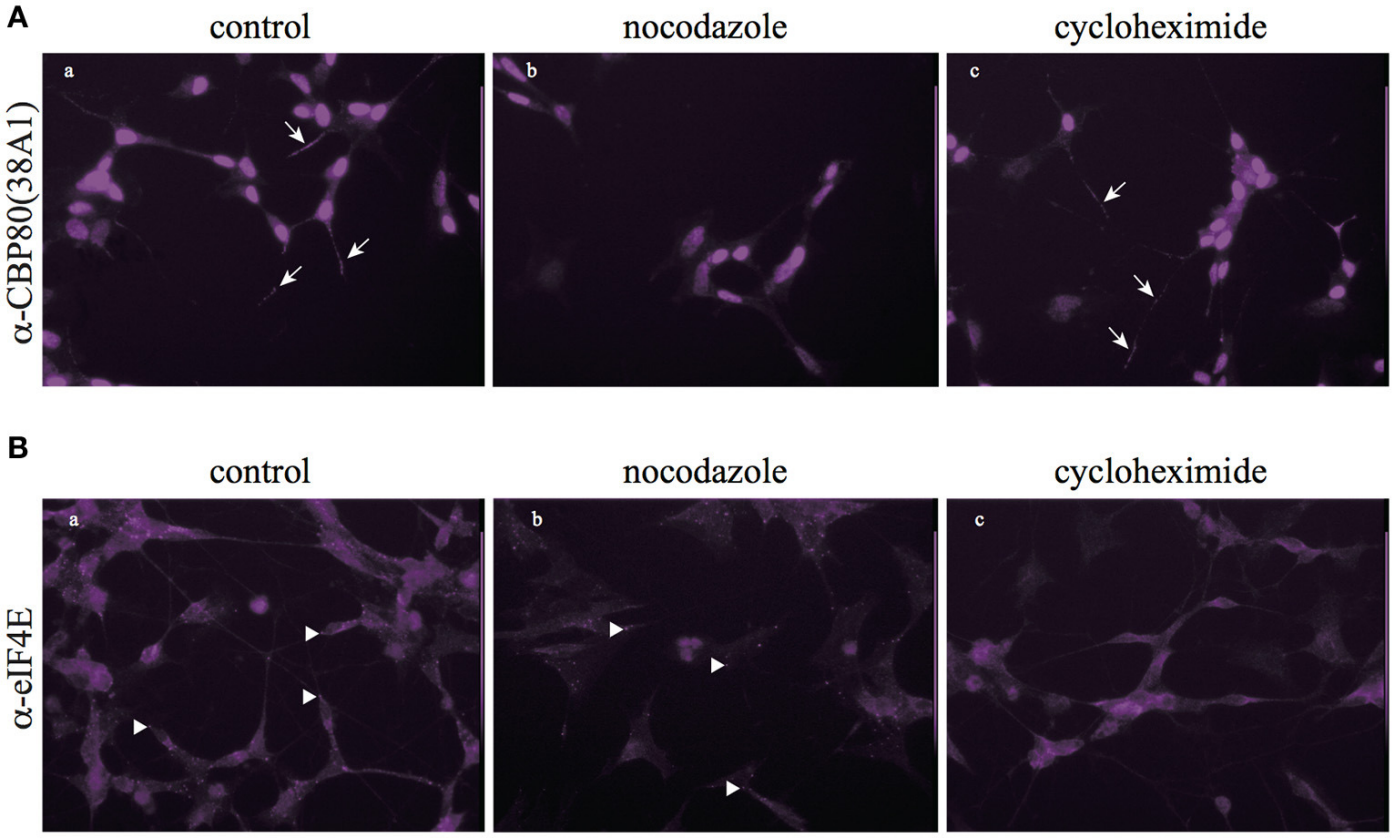
Nocodazole and cycloheximide differentially regulate CBP80- and eIF4E-RNPs. **(A)** Microimages of CBP80-staining of SH-SY5Y cells treated with nocodazole or cycloheximide; arrows point to CBP80-positive puncta; **(B)** Microimages of eIF4E staining of SH-SY5Y cells eIF4E treated with nocodazole or cycloheximide; arrowhead point to eIF4E-positive puncta.

In consistence with the biochemical fractionation analysis, when we treated living SH-SY5Y cells with nocodazole, CBP80-positive puncta diminished in the neuronal process while eIF4E-positive puncta remained largely intact (Figure 4), which suggests that the assembly and/or maintenance of CBP80-positive granules depend on microtubule polymerization, but that of eIF4E-positive granules do not. Cycloheximide treatment, however, selectively diminished the eIF4E-granules but not CBP80-granules from the neuronal process, suggesting that the assembly and/or maintenance of eIF4E granules are not dependent on the integrity of microtubule but on continuous translation (Figure 4). The distinct sensitivity of CBP80 granules and eIF4E granules to microtubule destabilizer (nocodazole) and translational inhibitor (cycloheximide) treatments suggest that the two types of localized granules are not only spatially segregated, but also behave and function distinctly in neuronal processes.

### CBP80-positive granules are distinct from P-bodies and are enriched with EJC components

**Figure 5:**
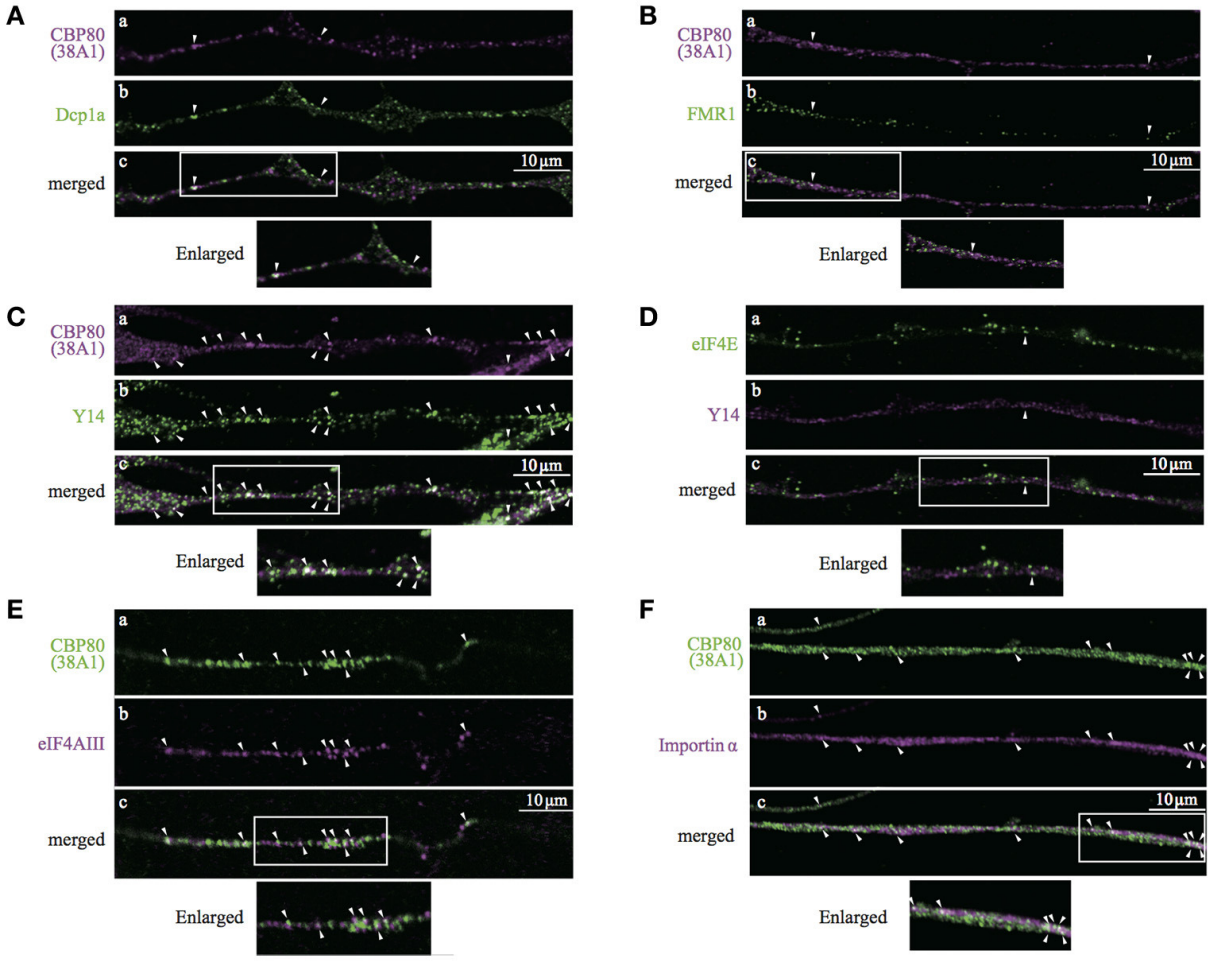
Confocal images of SH-SY5Y neuronal processes double-stained with antibodies against **(A)**, CBP80 (**a**, magenta) and Dcp1a (**b**, green); **(B)** CBP80 (**a**, magenta) and FMR1 (**b**, green); **(C)** CBP80 (**a**, magenta) and Y14 (**b**, green); **(D)** eIF4E (**a**, green) and Y14 (**b**, magenta), **(E)** CBP80 (**a**, green) and eIF4AIII (**b**, magenta), **(F)** CBP80 (**a**, green) and Importin α (**b**, magenta). Merged images are also presented as **(c)**. Scale bar, 10 μm. Enlarged images of white boxes are also presented at the bottom of the panels in each part.

Heterogeneous populations of translationally repressed RNA granules have been identified in neuronal processes, such as large transport granules, P-bodies, stress granules, and dendritic P-body-like structures. Resistance to cycloheximide and the role of CBP80 as cap recognition of newly synthesized mRNAs suggest that CBP-granules are also translationally silent. To identify the spatial relationship between the CBP80-granules and previously identified translationally repressed granules, we double-stained the differentiated SH-SY5Y cells with a variety of granule markers such as Dcp1a and FMR1, and analyzed colocalization with CBP80 in the neuronal processes. CBP80-granules and other types of granules resulted in poor colocalization (Figures 5A,B). These results indicate that CBP80-granules are distinct from previously described translationally silent granules.

The results of double-staining experiments suggest that CBP80 granules represent an early stage of RNPs containing newly synthesized mRNA. Thus, we co-stained differentiated SH-SY5Y neurons with Y14/RBM8A (a core component of EJC) and 38A1 and found that many but not all CBP80-positive granules also showed bright Y14 immunofluorescence (Figure 5C). In contrast, Y14 was poorly colocalized with eIF4E in neurites (Figure 5D). These results suggest that EJC remains associated with the mRNAs in these CBP80-positive granules. We then performed double staining of differentiated SH-SY5Y neurons with 38A1 and eIF4AIII, another core component of EJC. The immunostaining results indicate that many of CBP80 granules showed eIF4AIII signals (Figure 5E). These results support that mRNAs in CBP80-positive granules remain EJC-bound. We also tested whether Importin α is included in CBP80-bound granules, which may function for later replacement of CBC to eIF4E. The immunostaining results shown in Figure 5F demonstrate that some of CBP80-positive puncta showed Importin α staining signals. We have quantified colocalization of various protein markers on previously reported mRNPs and found that CBP80, EJC core components and Importin α showed significant co-localization (Supplemental Table 1). These results strongly suggest that CBP80 positive granules are EJC-containing mRNPs that are translationally repressed and have not yet undergone the very first translation. Since colocalization between Y14 and CBP80 but not Y14 and eIF4E was observed in the nucleus of SH-SY5Y cells (Supplemental Figure 2A), it is highly likely that CBP80 and Y14 double-positive granules are assembled in the nucleus and co-transported into the distal sites.

### CBP80-positive granules are present in the neuronal processes of hiPS-derived dopaminergic cells

**Figure 6:**
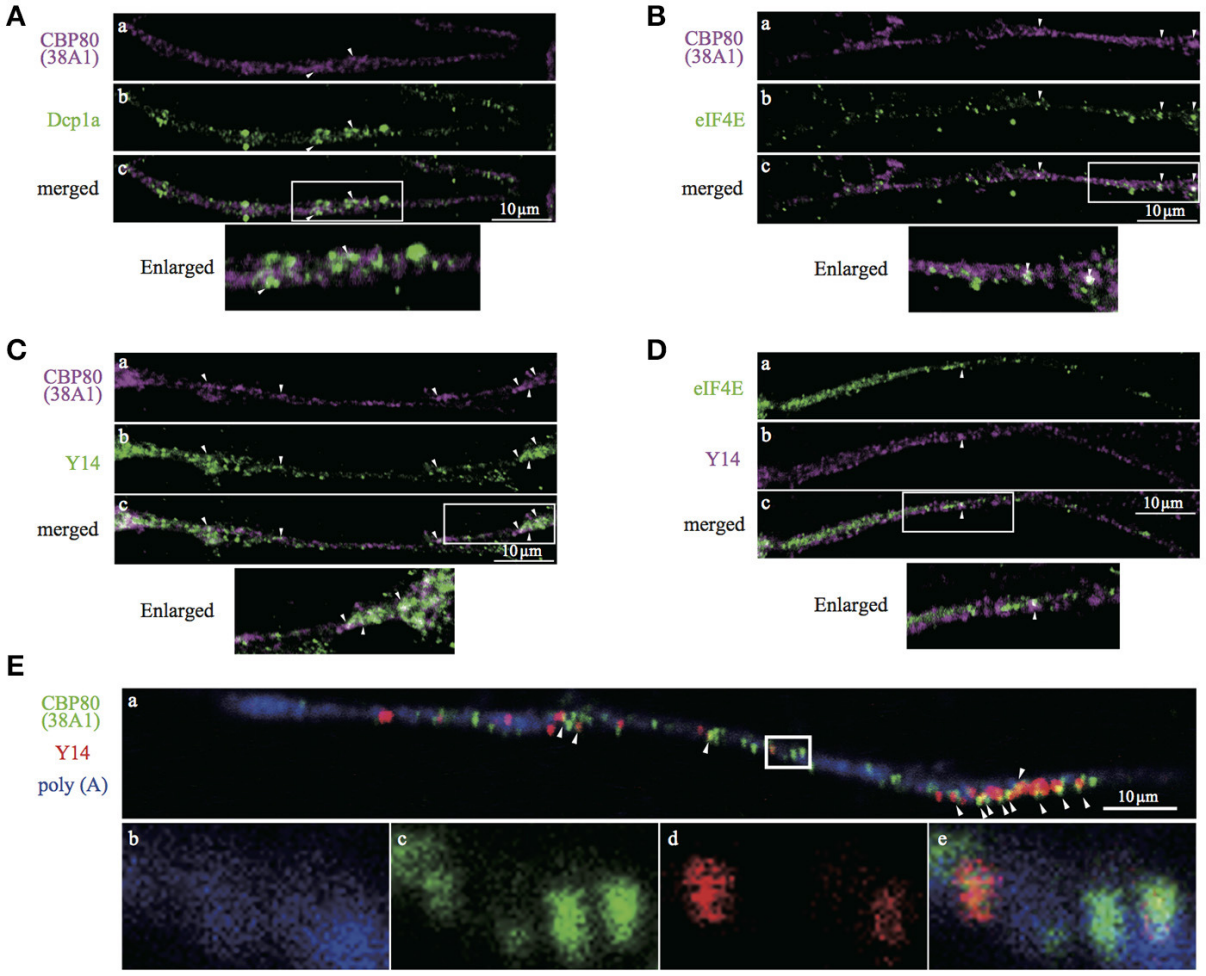
Confocal images of iPS-derived dopaminergic neurons double- or triple-stained with antibodies against. **(A)** CBP80 (**a**, magenta) and Dcp1a (**b**, green), **(B)** CBP80 (**a**, magenta) and eIF4E (**b**, green), **(C)** CBP80 (**a**, magenta) and Y14 (**b**, green), **(D)** eIF4E (**a**, green) and Y14 (**b**, magenta), **(E)** poly(A) RNA (**a,b**, blue), CBP80 (**a,c**, green), Y14 (**a,d**, red). Merged images are also presented as **(c)** and **(e)**. Scale bars, 10μm.

The characterization of CBP80 granules in SH-SY5Y cells suggests the presence of a population of mRNA in the human neuronal process that has not undergone the “very first translation.” To test this in primary human neurons, we turned to *in vitro* human iPS-derived neuronal cells. Immunostaining of differentiated cells with antibodies recognizing βIII-tubulin and tyrosine hydroxylase (TH) confirmed that 80–90% of the cultured cells were neurons (βIII-tubulin positive) of which 30% were dopaminergic neurons (TH positive). We repeated the double-staining experiments in iPS-derived neuronal cultures using combinations of antibodies (Dcp1a/CBP80, eIF4E/CBP80, Y14/CBP80 and eIF4E/Y14). Colocalization analysis showed consistent results with those in SH-SY5Y cells, confirming our findings in iPS-derived human neurons (Figure 6). In these cells, many of the Y14-positive granules overlapped with CBP80 granules in the nucleus (Supplemental Figure 2B), suggesting that CBP80 and Y14 containing granules are assembled in the nucleus and get transported to the cytoplasm. Finally we determined colocalization of CBP80, Y14 and poly(A) RNA in neurites of iPS-derived human neurons. As shown in Figure 6E, triple staining revealed a subset of granules that contain poly(A) RNA, CBP80 and Y14 (a and e). However, Y14 signal was absent in some granules containing both CBP80 and poly(A) RNA (b-e). These results strongly suggest the heterogeneity of CBP80 mRNP granules in terms of protein components, and likely mRNA species.

## Discussion

In this paper we describe distally localized mRNP granules that contain CBC and an EJC in the processes of human neuronal cells. CBC and EJC co-assemble with mRNAs in the nucleus and are exported to the cytoplasm as mRNPs. Since these factors are removed from mRNPs by a “very first translation,” it is likely the granules we observed have not been through the very first translation. Indeed, they are insensitive to cycloheximide treatment, and spatially segregated from the eIF4E-containing granules. These results strongly suggest that remodeling of mRNPs takes place during cytoplasmic transport including the exchange of the cap binding proteins. It is likely that CBP80 granules in neurites are transport granules, whereas eIF4E granules are storage and translating granules. CBP80-, but not eIF4E-granules mainly associated with microtubules, suggesting that a very first translation mediates the mRNP remodeling from transport granules to storage/translating granules.

How do CBC-granules escape the very first of translation? It has been demonstrated that both translation-independent and translation-dependent remodeling mechanisms can initiate the very first translation (Chiu et al., 2004). As for translation-independent mechanism, binding of Importin β to CBP80 NLS-Importin α complex destabilizes its binding to the cap structure (Gorlich et al., 1996; Sato and Maquat, 2009). Indeed, when differentiated SH-SY5Y cells were stained with anti-importin β antibody, a punctate staining pattern was identified throughout neurites (data not shown). One possible mechanism is that specific binders of CBP80 prevent Importin β from binding to Importin α-CBP80 complex during transport in neurites. The possible mechanism for translation-dependent step is to inhibit the binding of translation initiation factor eIF4G to CBP80. In this case, unidentified factor(s) that contain similar interacting domains of CBP80 and eIF4G may block their interaction. These mechanisms are not mutually exclusive. Identification of specific binders to CBP80/Importin α complexes from neuronal cells is required to find candidates for such factors.

In addition to regulations through NMD and very first translation, translation repression mechanisms have been demonstrated by inhibiting any of the three main translation steps: initiation, elongation and termination. For repression of translational initiation, specific binding proteins to prevent eIF4G binding to eIF4E such as 4EBP, Maskin, neuroguidin, and CYFIP1 have been identified that can be regulated by mTOR signaling pathways and also cytoplasmic polyadenylation element-binding protein (CPEB) (for review, Wang et al. 2010). Noncoding RNA BC1, has also been shown to bind to eIF4A, blocking its helicase activity (Lin et al., 2008). We have tested 4EBP localization in SH-SY5Y neurites, and found that 4EBP co-localized with eIF4E, but not with CBP80 (data not shown). We also demonstrated that decapping enzyme Dcp1a, a P-body marker (Fenger-Gron et al., 2005; Lykke-Andersen and Wagner, 2005), does not co-localize with CBP80-RNPs either. Therefore, it is unlikely that CBP80 bound mRNPs utilize the same mechanisms that eIF4E granules employ for translation repression during cytoplasmic transport. Other mechanisms for translation repression are, for example, Pumilio family binding to 3′-UTRs and promoting deadenylation, FMR1 protein binding to G-quartet of mRNAs and inhibiting translation, microRNA such as miR-134 and miR-138 blocking translation of LIMK and APT1F at synapse (Darnell et al., 2004; Schratt et al., 2006; Goldstrohm et al., 2008; Siegel et al., 2009; Maurin et al., 2014). We found that FMR1 and CBC-granules barely co-localized (Figure 4, Supplemental Table 1), suggesting that FMR1 is not involved in the translational repression of CBP80 granules. Whether other known translational repressors and transport factors are co-localized with CBP80 and Y14 in neuritis needs further investigations.

It was reported by di Penta et al. that dendritic LSm1 mRNP granules contain CBP80 and that they shift into spines upon stimulation of glutamatergic receptors (di Penta et al., 2009). Recently it was also reported that Staufen2 containing mRNP granules contain CBP80 in neurons (Fritzsche et al., 2013; Heraud-Farlow et al., 2013). CBP80-containing granules may be heterogenic. Indeed, not all of CBP80-positive mRNPs had Y14 signals (Figure 6). We have to compare both RNA and protein constituents of CBP80/Staufen2 granules and CBP80/Lsm1 granules with the CBP80/EJC granules we report here in order to characterize the heterogeneity.

Our results strongly suggest a very first translation mediated mRNP remodeling during cytoplasmic transport. It is highly likely once exchange of cap binding proteins takes place, eIF4E-bound mRNPs can no longer be transported but are either stored or translated locally. It is not known how this process is regulated spatiotemporally. Further detailed analysis of the CBP80/Y14 granules will contribute to understanding of transport, local translation and degradation of mRNAs in neurons.

## Author contributions

DW, KN, MO, and NK conceived and designed the experiments. DW, KN, CM, AK, MarH, MK, and NK performed the experiments. DW, KN, MK, MarH, KC, S-IT, MO, and NK analyzed the data. DW, KN, CM, AK, MarH, MK, MO, and NK contributed reagents/materials/analysis tools. DW, KN, MarH, MO, and NK wrote the paper. NK took the primary responsibility for the final content. DW, KN, CM, AK, MarH, MK, MasH, KC, S-IT, MO, and NK read and approved the final manuscript.

### Conflict of interest statement

The authors declare that the research was conducted in the absence of any commercial or financial relationships that could be construed as a potential conflict of interest.
