# Supplementary figures and images for: Transport Granules Bound with Nuclear Cap Binding Protein and Exon Junction Complex Are Associated with Microtubules and Spatially Separated from eIF4E Granules and P Bodies in Human Neuronal Processes
Source: Front Mol Biosci. 2017 Dec 22;4:93. doi: 10.3389/fmolb.2017.00093 (PMC5744441; doi:10.3389/fmolb.2017.00093)

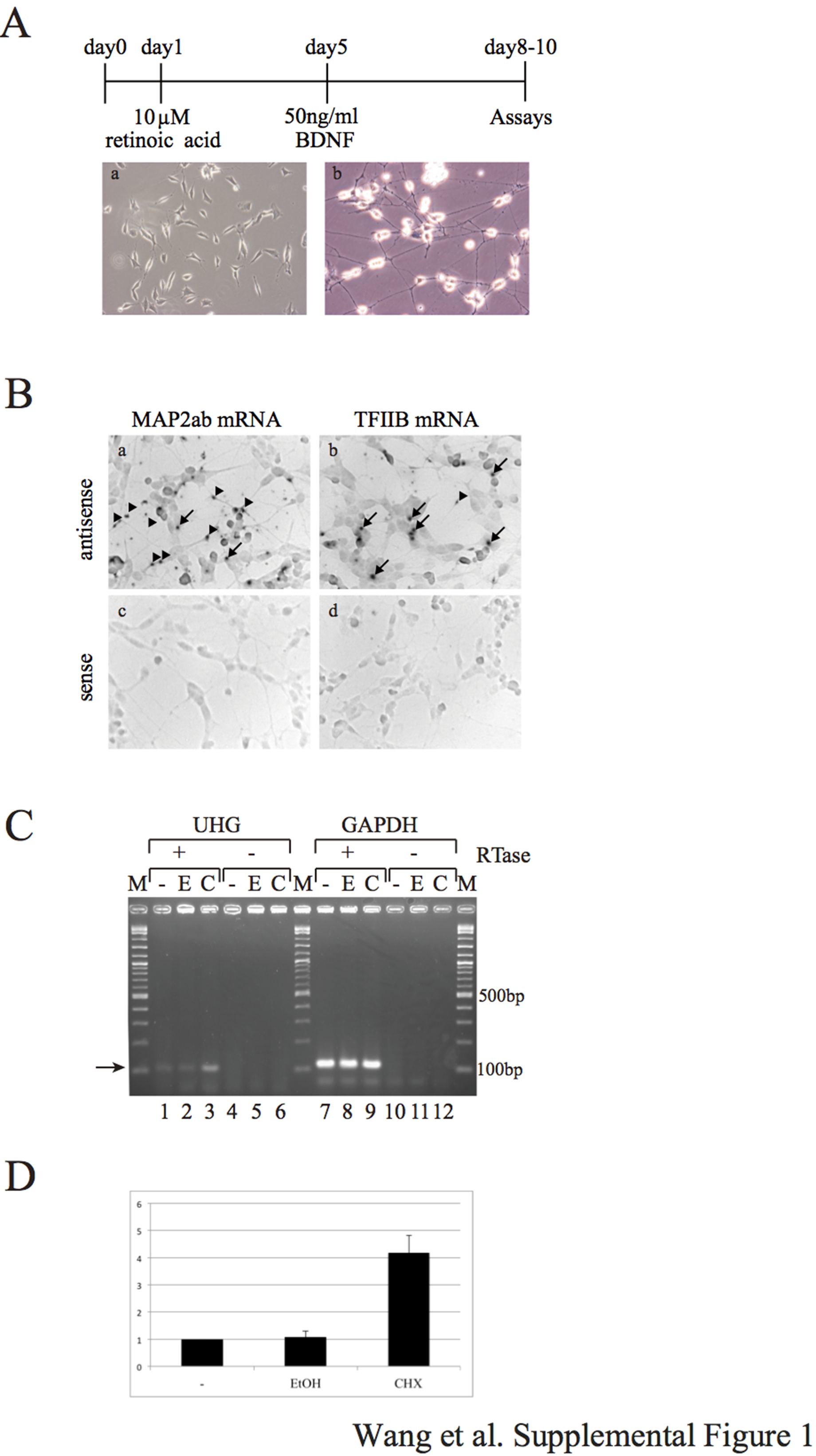

Supplement: Supplementary file 1 [file Image1.TIFF]

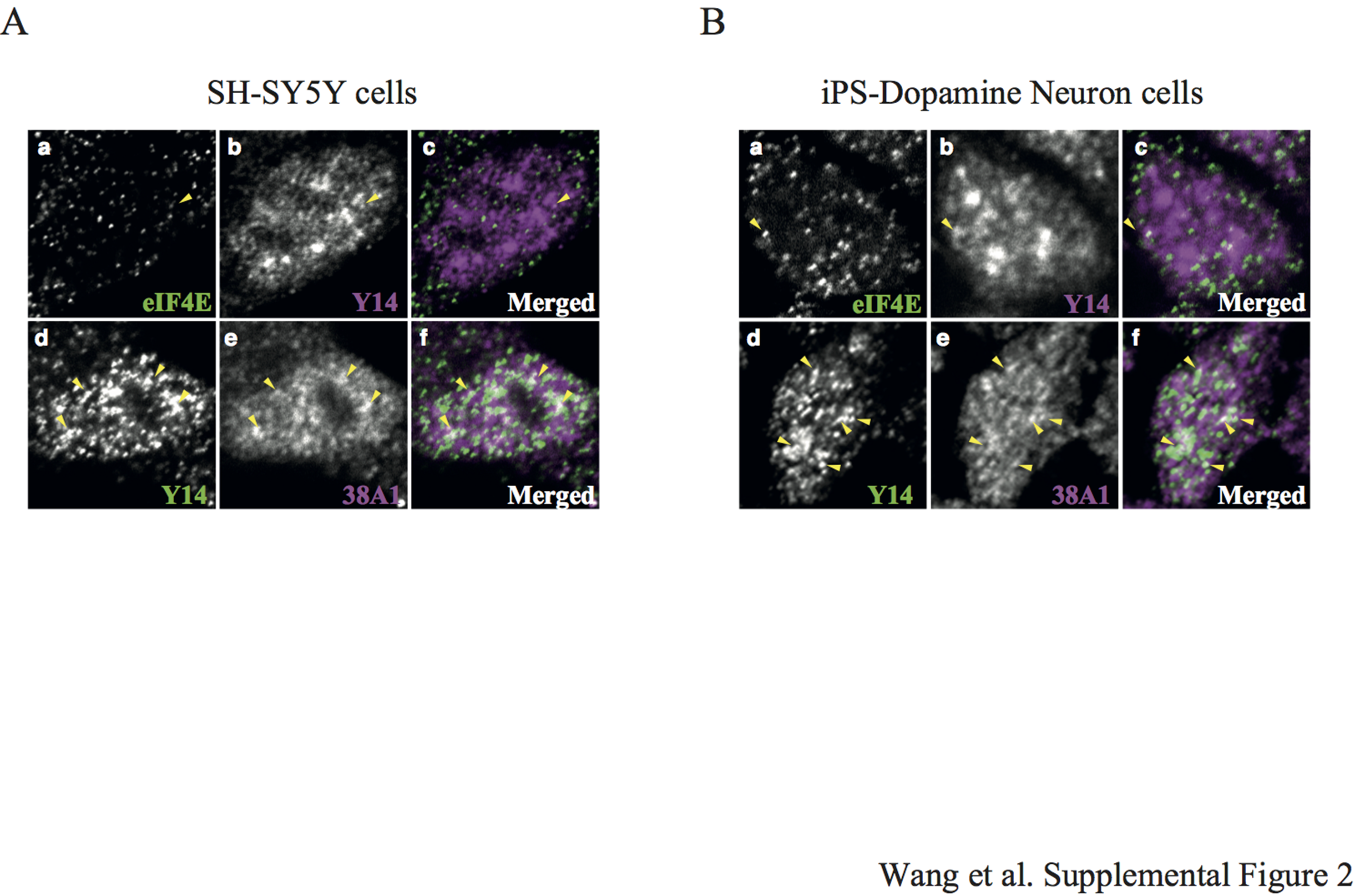

Supplement: Supplementary file 2 [file Image2.TIFF]

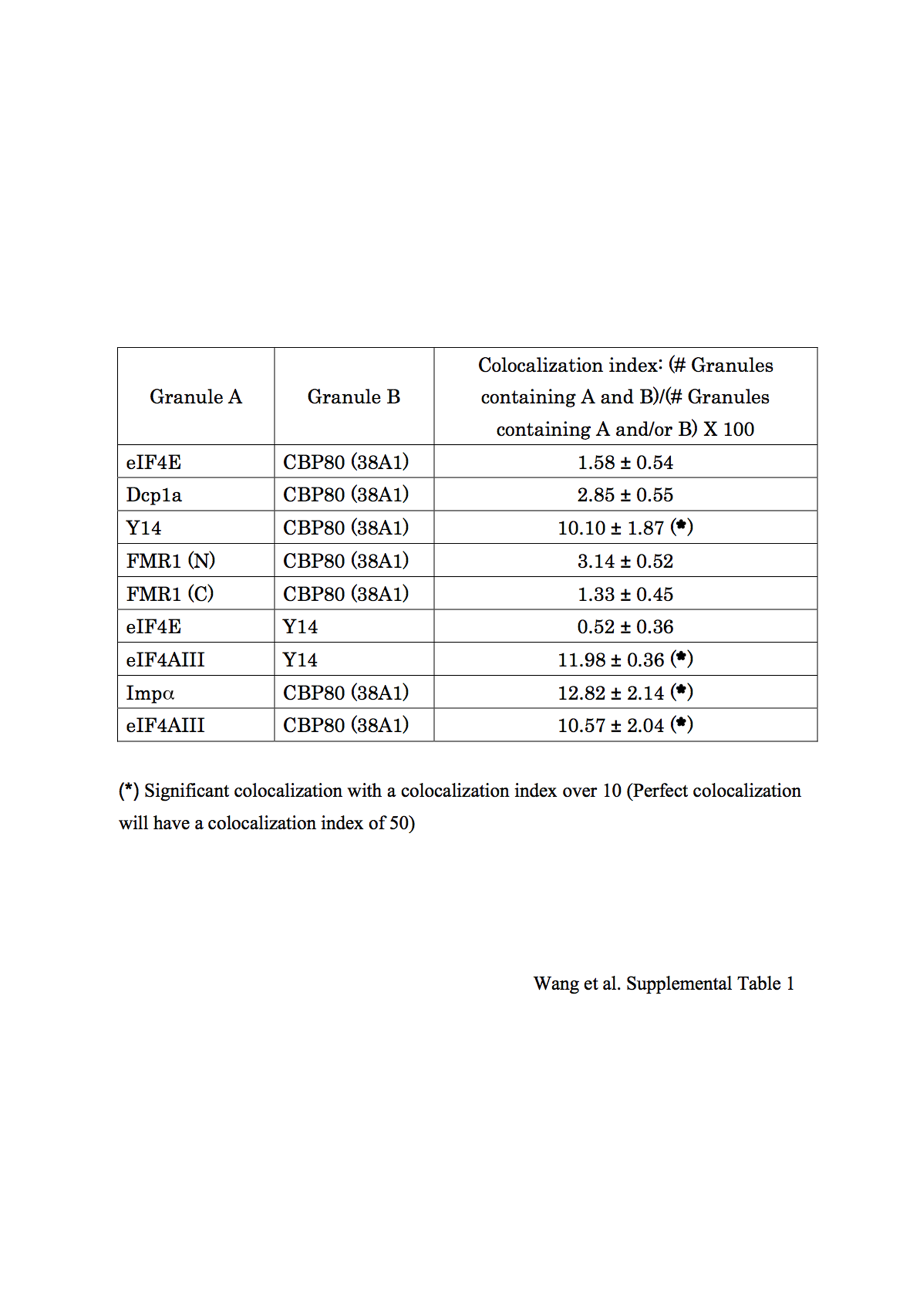

Supplement: Supplementary file 3 [file Table1.TIFF]
