# Supplementary material for: Transport Granules Bound with Nuclear Cap Binding Protein and Exon Junction Complex Are Associated with Microtubules and Spatially Separated from eIF4E Granules and P Bodies in Human Neuronal Processes
Source: Front Mol Biosci. 2017 Dec 22;4:93. doi: 10.3389/fmolb.2017.00093 (PMC5744441; doi:10.3389/fmolb.2017.00093)
Supplement: Supplementary file 4 [file SupplementaryMaterial.pdf]

Supplemental Figure legends

Supplemental Figure 1. Characterization of SH-SY5Y cells as a model cell for mRNP transport, localization and NMD.

(A) A procedure for SH-SY5Y cell differentiation. The microscopic image of undifferentiated (a) and differentiated (b) SH-SY5Y cells are also shown. (B) Detection of localized mRNA in neuronal processes of differentiated SH-SY5Y cells by *in situ* hybridization. Arrows indicate signals in cell bodies, while arrowheads point to signals in neuronal processes. (C) Nonsense mediated mRNA decay activity in differentiated SH-SY5Y cells. Cells were treated with ethanol (lane E) or cycloheximide (C) or without treatment (-) and total RNAs were recovered. RT-PCR analyses were carried out for a natural NMD substrate UHG and an internal control GAPDH. RT-PCR reaction without a reverse transcriptase (RTase) serves as a negative control. The band corresponding to UHG product is indicated by an arrow. (D) Relative levels of UHG mRNA compared to GAPDH mRNA in non-treated (-), ethanol treated (EtOH) and cycloheximide treated (CHX) cells measured by quantitative RT-PCR.

Supplemental Figure 2. Confocal images of nuclei region of SH-SY5Y cells (A) and iPS-dopaminergic neurons (B) double-stained with antibodies against eIF4E (green) and Y14 (magenta), Y14 (green) and CBP80 (38A1)

(magenta). Yellow arrowheads point to concentrations of both immunofluorescence in the nuclei. Scale bars, 5μm.

Supplemental Table 1. Colocalization analysis of RNA granules in SH-SY5Y neuronal processes.
